# Successes and Challenges in the Elimination of Congenital Syphilis in Brazil: Evidence of Progress and Persistent Challenges

**DOI:** 10.1155/jotm/6589431

**Published:** 2026-07-27

**Authors:** Iaron Leal Seabra, Nyla Lauane Costa de Almeida, Bianca Rafaele Souza Trindade, Arthur da Silva Costa Pedroza, Thayse Moraes de Moraes, Glenda Roberta Oliveira Naiff Ferreira, Eliã Pinheiro Botelho

**Affiliations:** ^1^ Graduate Program in Nursing, Federal University of Pará, Belém, Pará, Brazil, ufpa.br; ^2^ Department of Community Health, State University of Pará, Belém, Pará, Brazil, uepa.br

## Abstract

**Background:**

Brazil has implemented multiple policies targeting congenital syphilis (CS); however, evaluations of their effectiveness remain limited. This study aimed to analyze temporal trends in annual rates of gestational syphilis (GS) and CS from 2011 to 2022 across Brazil and its regions. It was hypothesized that effective prenatal care would enable early GS detection and that appropriate treatment of pregnant patients and their partners would reduce CS detection rate.

**Methods:**

This temporal study used GS and CS notification data from all Brazilian municipalities. National and regional annual rates were analyzed using the joinpoint regression model. Annual percent change (APC), average APC (AAPC), 95% confidence intervals, and *p* values were calculated.

**Results:**

A total of 586,855 new GS cases and 272,491 CS cases were reported during the study period. GS rates increased throughout Brazil and all regions, accompanied by a decline in APC values. For CS, rates achieved overall stabilization in Brazil as well as the North, Midwest, and South regions, whereas the Southeast and Northeast regions experienced continuous growth with a reduced APC from 2015 and 2017, respectively.

**Conclusion:**

Although CS trends stabilized or increased at reduced APC values over time, rates remain high. Additional efforts are required to eliminate the disease, including earlier initiation of prenatal care and improved prevention, diagnosis, and treatment of syphilis in pregnant patients and their partners.

## 1. Introduction

The elimination of congenital syphilis (CS) by 2030, as proposed by the World Health Organization (WHO) in the Sustainable Health Agenda for the Americas 2018–2030 [1], remains a distant target. As of 2021, only eight countries had achieved CS elimination certification: Anguilla, Antigua and Barbuda, Bermuda, the Cayman Islands, Cuba, Dominica, Montserrat, Saint Kitts, and Nevis [2]. Globally, approximately 700,000 cases of gestational syphilis (GS) and 700,000 cases of CS were reported in 2022 with an estimated 390,000 adverse birth outcomes [3].

Brazil, a signatory to the 2030 Agenda [4], has implemented multiple policies to expand access to prenatal care and to strengthen prevention, diagnosis, and treatment of syphilis. These initiatives include the implementation of the Stork Network to improve maternal and child health; incorporation of rapid tests for HIV, syphilis, and hepatitis B and C into routine prenatal care; decentralization of rapid testing and condom distribution within the primary health care (PHC) network; publication of the first Clinical Protocol and Therapeutic Guidelines for the Prevention of Vertical Transmission of HIV, Syphilis, and Viral Hepatitis; the Agenda of Strategic Actions for the Reduction of Congenital Syphilis in Brazil; and the development of certification guidelines for eliminating vertical transmission of syphilis and/or HIV [5, 6].

Despite improvements in prenatal services in Brazil [7], the expected pattern of increased GS detection accompanied by reduced CS detection rates has not been observed. Data from the Brazilian Epidemiological Bulletin indicate an increase in the incidence rates of acquired syphilis (AS), GS, and CS between 2013 and 2022 [8]. Supporting Table 1 presents the AS, GS, and CS rates for 2012 and 2023, along with the percentage increase from 2012 to 2023.

Given these findings, it is hypothesized that effective prenatal care should enable early GS detection and reduce CS rates. However, studies evaluating temporal trends in GS and CS concurrently across Brazil remain limited. Therefore, this study conducted a time‐series analysis of GS and CS across the entire country and its regions from 2011 to 2022. Time‐series analysis is a robust method for identifying temporal changes and associating them with health policies and social determinants [9]. To our knowledge, comparative analyses across Brazilian regions remain scarce.

## 2. Methods

This time‐series study used secondary data from compulsory notifications of GS and CS across all Brazilian regions. Data were obtained from DATASUS [10] and included year of diagnosis, municipality of residence of pregnant patients, and the number of live births per municipality. Only reported GS and CS cases involving pregnant patients residing in Brazil were included. All variables were aggregated by region. Data were manually curated, and duplicate entries were removed.

Annual detection rates for GS and CS were calculated using the Brazilian Ministry of Health methodology, in which the number of CS and GS cases was divided by the number of live births for the corresponding year and location, multiplied by 1000 [8].

Temporal trends in GS and CS rates were analyzed using joinpoint regression analysis via the Joinpoint Trend Analysis software, Version 5.2.0 (National Cancer Institute, Bethesda, MD, USA). Rates were treated as dependent variables and years as the independent variable. The regression models were fitted under default software specifications, utilizing the Monte Carlo permutation method (4999 permutations) for model selection, assuming constant error variance and independent observations. A maximum of two joinpoints was permitted, and the final models selected two joinpoints. Rates were log‐transformed prior to analysis to estimate the annual percent change (APC) and average APC (AAPC). Corresponding 95% confidence intervals (95% CI) were calculated using the empirical quantile method [11].

Trends were classified as increasing or decreasing when APC values were positive or negative, respectively, with *p* ≤ 0.05. APC values with *p* > 0.05 were considered stable. All analyses used a 95% CI, and AAPC values were calculated. Models were generated for each region and for Brazil overall to allow comparative evaluation of temporal trends.

According to Resolution No. 510 of 2016 from the Brazilian Health Council, studies using publicly available, nonidentifiable data do not require Ethics Committee approval.

## 3. Results

During the study period, 586,855 GS cases and 272,491 CS cases were reported in Brazil. Based on AAPC estimates, GS rates showed continuous upward trends nationally and across all regions (Figure 1A, C, E, G, I, K; Table 1). However, a reduced APC was observed after 2017 in Brazil, as well as in the North, Southeast, and South regions (2011–2018–Brazil: APC = 23.78, *p* < 0.01; 2018–2022–Brazil: APC = 10.78, *p* < 0.01; 2011–2017–North: APC = 23.19, *p* < 0.01; Southeast: APC = 23.33, *p* < 0.01; 2017–2022–North: APC = 14.15, *p* < 0.01; Southeast: APC = 11.43, *p* < 0.01; 2011–2016–South: APC = 37.38, *p* < 0.01; 2016–2022–South: APC = 10.05, *p* < 0.01). In the Midwest, GS rates rose from 2011 to 2018 before stabilizing through 2022 (2011–2018: APC = 20.09, *p* < 0.01; 2018–2022: APC = 10.98, *p* = 0.07). In contrast, the Northeast followed nonlinear upward trajectory, with the APC peaking between 2015 and 2018 (2011–2015: APC = 15.64, *p* < 0.01; 2015–2018: APC = 33.5, *p* < 0.01; 2018–2022: APC = 10.04, *p* < 0.01).

FIGURE 1Temporal trend analysis of GS and CS in Brazil. GS detection trends for Brazil and its regions are presented in panels (A, C, E, G, I, and K), where CS trends are shown in panels (B, D, F, H, J, and L). Red squares indicate GS and CS detection rates. ^∗^ *p* < 0.01.

**Figure:**
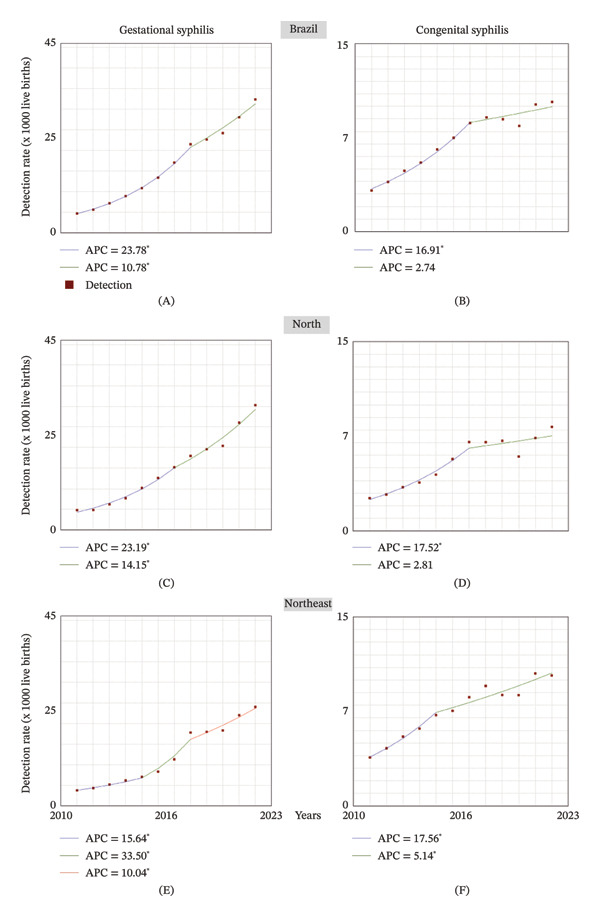

**Figure:**
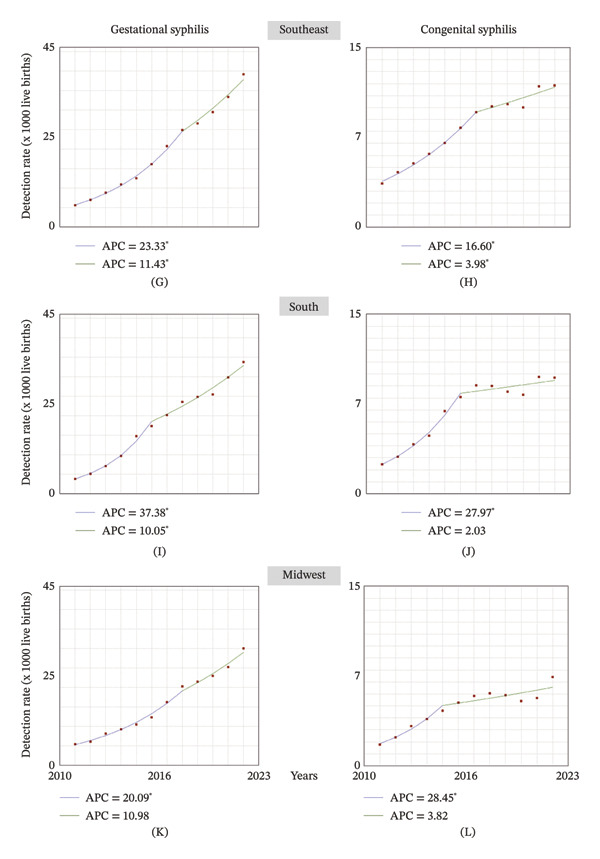

**TABLE 1:** Temporal trend analysis of GS detection rates in Brazil and its regions, 2011–2022.

Region	Period	APC (95% CI)	*p* value	AAPC (95% CI)	*p* value
Brazil	2011–2018	23.78 (22.48 to 25.27)	< 0.01	18.88 (18.14 to 19.57)	< 0.01
	2018–2022	10.78 (8.04 to 13.38)	< 0.01		

North	2011–2017	23.19 (20.32 to 30.97)	< 0.01	18.99 (17.40 to 20.64)	< 0.01
	2017–2022	14.15 (6.23 to 17.54)	< 0.01		

Northeast	2011–2015	15.64 (6.71 to 19.87)	< 0.01	18.10 (16.46 to 19.38)	< 0.01
	2015–2018	33.50 (25.38 to 39.16)	< 0.01		
	2018–2022	10.04 (3.29 to 13.91)	< 0.01		

Midwest	2011–2018	20.09 (17.86 to 29.39)	< 0.01	16.69 (14.29 to 19.03)	< 0.01
	2018–2022	10.98 (−3.49 to 15.78)	0.07		

Southeast	2011–2017	23.33 (21.57 to 25.97)	< 0.01	18.85 (17.47 to 19.99)	< 0.01
	2017–2022	11.43 (4.67 to 15.01)	< 0.01		

South	2011–2016	37.38 (34.46 to 40.34)	< 0.01	21.72 (20.73 to 22.65)	< 0.01
	2016–2022	10.05 (8.12 to 11.82)	< 0.01		

Similar to GS rates, the AAPC indicated a continuous upward trend in CS rates, whereas the APC analysis identified two distinct temporal segments across Brazil and its regions (Figure 1B, D, F, H, J, L; Table 2). Overall, CS rates initially escalated before stabilizing in Brazil (Figure 1B), and the North (Figure 1D), Midwest (Figure 1L), and South regions (Figure 1J) (2011–2017–Brazil: APC = 16.91, *p* < 0.01; North: APC = 17.52, *p* < 0.01; 2017–2022–Brazil: APC = 2.74, *p* = 0.12; North: APC = 2.81, *p* = 0.34; 2011–2015–Midwest: APC = 28.45, *p* < 0.01; 2015–2022–Midwest: APC = 3.82, *p* = 0.17; 2011–2016–South: APC = 27.97, *p* < 0.01; 2016–2022–South: APC = 2.03, *p* = 0.2). In contrast, the Northeast (Figure 1F) and Southeast (Figure 1H) regions exhibited continuous growth, albeit with a marked decrease in APC in the later years (Southeast: 2011–2017: APC = 16.60, *p* < 0.01; 2017–2022: APC = 3.98, *p* < 0.01; Northeast: 2011–2015: APC = 17.56, *p* < 0.01; 2015–2022: APC = 5.14, *p* < 0.01). Although APC patterns for GS and CS were broadly similar, inflection points occurred earlier for CS in the Midwest and Northeast regions (Midwest: GS = 2018–2022, CS = 2015–2022; Northeast: GS = 2018–2022, CS = 2015–2022).

**TABLE 2:** Temporal trend analysis of CS detection rates in Brazil and its regions, 2011–2022.

Region	Period	APC (95% CI)	*p* value	AAPC (95% CI)	*p* value
Brazil	2011–2017	16.91 (14.37 to 20.28)	< 0.01	10.24 (9.0 to 11.42)	< 0.01
	2017–2022	2.74 (−1.13 to 5.68)	0.12		

North	2011–2017	17.52 (13.69 to 24.93)	< 0.01	10.59 (8.29 to 12.74)	< 0.01
	2017–2022	2.81 (−5.55 to 7.25)	0.34		

Northeast	2011–2015	17.56 (13.17 to 25.33)	< 0.01	9.50 (8.24 to 10.68)	< 0.01
	2015–2022	5.14 (2.40 to 6.98)	< 0.01		

Midwest	2011–2015	28.45 (18.14 to 54.09)	< 0.01	12.18 (8.96 to 16.05)	< 0.01
	2015–2022	3.82 (−3.21 to 7.91)	0.17		

Southeast	2011–2017	16.60 (14.68 to 18.82)	< 0.01	10.69 (9.83 to 11.51)	< 0.01
	2017–2022	3.98 (1.29 to 6.21)	< 0.01		

South	2011–2016	27.97 (22.92 to 33.63)	< 0.01	13.09 (11.49 to 14.66)	< 0.01
	2016–2022	2.03 (−1.52 to 5.15)	0.2		

## 4. Discussion

The findings indicate a sustained increase in GS cases, although the rate of growth was lower in the later years of the series. Although the ecological design of the present study does not allow causal inferences regarding the effect of public policies on GS trends, the observed temporal pattern may coincide with the implementation of public health initiatives in Brazil, including the decentralization of rapid testing and condom distribution within the PHC network in 2012, which aimed to expand access to preventive measures, and the implementation of the Stork Network in 2011, which expanded prenatal care coverage [5]. Furthermore, while Brazil’s adoption of the United Nations 2030 Agenda for Sustainable Development in 2016 established critical elimination targets [4], the subsequent implementation of the Strategic Action Agenda for the Reduction of Syphilis, renewed in 2017 and 2020, may have contributed to strengthening health system responses and slowing the expansion of the epidemic [12].

CS rates exhibited two temporal patterns, with stabilization observed in the second period, except in the Northeast and Southeast regions. This stabilization may be associated with improvements in prenatal care, including mandatory syphilis testing at the first prenatal visit and during the third trimester since 2012 [5], as well as the introduction of partner prenatal care in 2017 [13], which facilitated earlier diagnosis among pregnant women and their partners. Additionally, a 2016 national campaign promoted early prenatal care, expanded rapid testing, and enhanced treatment access for pregnant women and their partners, alongside ongoing professional training [14].

The expansion of Family Health Strategy (FHS) coverage may also have contributed to stabilizing CS trends or reducing APC. As the primary entry point to the Unified Health System (SUS), the FHS plays a critical role in early identification of pregnant women and their partners, thereby supporting timely prenatal care [15]. Between 2006 and 2016, FHS coverage increased nationwide, particularly in the Southeast and North regions (annual variation: Brazil = 8.4%, North = 10.3%, Northeast = 4.6%, Midwest = 7.9%, Southeast = 11.8%, and South = 10.1%) [15]. This expansion aligns with findings from the Program for Improving Access and Quality, which reported increases between 2012 and 2017 in the average number of prenatal visits per pregnant woman (North: 168%; Northeast: 220%; Midwest: 271%; Southeast: 444%; South: 284%) and in the proportion initiating care during the first trimester (North: 77%; Northeast: 146%; Midwest: 175%; Southeast: 189%; South: 114%) [7]. However, these improvements did not translate into proportional reductions in syphilis burden. Even in regions with stable CS trends (North, South, and Midwest), rates remained elevated.

In contrast, the Northeast and Southeast regions demonstrated persistent upward CS trends, although with lower APC in the second period. These data suggest structural gaps in prenatal in these regions. In 2023, only 34.5% of GS cases in the Northeast were diagnosed during the first trimester, and only 28.1% of partners received treatment. States such as Pernambuco, Sergipe, and Rio Grande do Norte reported high proportions of GS cases without prescribed treatment (11.2%, 12.5%, and 10.8%, respectively). In the Southeast, although most GS cases were diagnosed early and 93% received treatment, only 35.7% of partners were treated [16].

Studies in Northeast and Southeast regions of Brazil suggest that limited syphilis screening, inadequate treatment of pregnant women, and lack of partner treatment remain important barriers to the control of congenital syphilis. A temporal study in Rio de Janeiro, Southeast region, identified an upward CS trend between 2021 and 2023, associated with reduced syphilis testing during the second trimester (first vs. second trimester: 82.7% vs. 52.6%), high rates of late GS diagnosis (33%), and low rates of adequate treatment (13.4%) [17]. Similarly, a retrospective study in the Northeast (2014–2018) reported that although 79.8% of pregnant women diagnosed with GS received prenatal care, 59.2% of them and 59.5% of their partners were inadequately treated, contributing to a sustained risk of reinfection [18].

Overall, even in regions that attained stable CS trends, persistently high rates underscore the substantial challenges to elimination. Nevertheless, progress is evident. In municipalities of the Federal District, the implementation of a plan aiming for continuous care for the pregnant woman, training of health workers, tracking of sexual partners’ tests, and a better referral system for infants exposed to syphilis resulted in a CS decrease of 37% from 2021 to 2023 [19]. Furthermore, in 2023, two Brazilian municipalities achieved CS elimination certification, and 46 others were recognized for best practices [16].

## 5. Conclusion

Joinpoint analysis demonstrated an increasing GS trend with deceleration in the most recent period. CS trends increased initially, followed by stabilization in Brazil and in the North, South, and Midwest regions. In contrast, the Northeast and Southeast regions exhibited two increasing trends, with a reduced APC in the latter period gaps in prenatal care. These findings indicate that CS elimination remains a major public health challenge and highlight the need to improve prenatal care quality.

## Funding

This study received no external funding.

## Conflicts of Interest

The authors declare no conflicts of interest.

## Supporting Information

Additional supporting information can be found online in the Supporting Information section.

## Supporting information


**Supporting Information** Supporting Table 1 presents the annual AS, GS, and CS rates in Brazil and its regions for 2012 and 2023, along with the overall percentage changes over the 12‐year period. Data were extracted from the 2024 Epidemiological Bulletin [8]. These supporting data provide additional context for interpreting the epidemiological trends of syphilis in Brazil.

## Data Availability

All data are publicly available through DATASUS: https://datasus.saude.gov.br/informacoes-de-saude-tabnet/.
