# Supplementary material for: Successes and Challenges in the Elimination of Congenital Syphilis in Brazil: Evidence of Progress and Persistent Challenges
Source: J Trop Med. 2026 Jul 27;2026:6589431. doi: 10.1155/jotm/6589431 (PMC13403759; doi:10.1155/jotm/6589431)
Supplement: Supplementary file 1 — Supporting Information Supporting Table 1 presents the annual AS, GS, and CS rates in Brazil and its regions for 2012 and 2023, along with the overall percentage changes over the 12‐year period. Data were extracted from the 2024 Epidemiological Bulletin [8]. These supporting data provide additional context for interpreting the epidemiological trends of syphilis in Brazil. [file JOTM-2026-6589431-s001.docx]

Suppl. Table 1 – Detection rates and overall percentage change of acquired, gestational, and congenital syphilis in Brazilian regions, 2013–2022.

| Syphilis category | Region | Year | | Overall change (%) |
| --- | --- | --- | --- | --- |
|  |  | 2013 | 2022 |  |
| Acquired syphilis  (x100,000 inhabitants) | North | 8.7 | 86.3 | 891.95 |
|  | Northeast | 6.1 | 55.4 | 808.19 |
|  | Midwest | 11.8 | 96.6 | 718.64 |
|  | Southeast | 31.6 | 112.9 | 257.27 |
|  | South | 21.5 | 151.2 | 603.25 |
| Gestatational syphilis  (x1,000 live births) | North | 6.2 | 30.4 | 390.32 |
|  | Northeast | 5.2 | 24.0 | 361.53 |
|  | Midwest | 8.2 | 30.1 | 267.07 |
|  | Southeast | 8.8 | 39.2 | 345.45 |
|  | South | 7.3 | 33.8 | 363.01 |
| Congenital syphilis  (x1,000 live births) | North | 3.5 | 8.4 | 140.00 |
|  | Northeast | 5.5 | 10.3 | 87.27 |
|  | Midwest | 3.3 | 7.4 | 124.24 |
|  | Southeast | 5.3 | 11.8 | 122.64 |
|  | South | 4.1 | 9.7 | 136.58 |
